# INTERACT: A comprehensive approach to assess urban form interventions through natural experiments

**DOI:** 10.1186/s12889-018-6339-z

**Published:** 2019-01-10

**Authors:** Yan Kestens, Meghan Winters, Daniel Fuller, Scott Bell, Janelle Berscheid, Ruben Brondeel, Michael Cantinotti, Geetanjali Datta, Lise Gauvin, Margot Gough, Karen Laberee, Paul Lewis, Sébastien Lord, Hui ( Henry) Luan, Heather McKay, Catherine Morency, Nazeem Muhajarine, Trisalyn Nelson, Callista Ottoni, Zoe Poirier Stephens, Caitlin Pugh, Gabrielle Rancourt, Martin Shareck, Joanie Sims-Gould, Meridith Sones, Kevin Stanley, Benoit Thierry, Calvin Thigpen, Rania Wasfi

**Affiliations:** 1 École de Santé Publique de l’Université de Montréal / Centre de recherche du CHUM, Pavillon S, Tour St–Antoine – 850 St–Denis – S03–280 –, Montreal, QC H2X 0A9 Canada; 2 Simon Fraser University, 8888 University Drive, Burnaby, BC V5A 1S6 Canada; 3 Memorial University of Newfoundland, 230 Elizabeth Avenue, St. John’s, NF A1C 5S7 Canada; 4 University of Saskatchewan, 105 Administration Place, Saskatoon, SK S7N 5A2 Canada; 5 Université du Québec à Trois-Rivières, 3351 Boulevard des Forges, Trois-Rivières, QC G9A 5H7 Canada; 6 University of British Columbia, 2329 West Mall, Vancouver, BC V6T 1Z4 Canada; 7 Polytechnique Montréal, 2900 Edouard Montpetit Blvd, Montreal, QC H3T 1J4 Canada; 8 Arizona State University, PO Box 875302, Tempe, AZ 85287-5302 USA; 9 University of Toronto, 155 College Street, Toronto, ON M5T 1P8 Canada

**Keywords:** Natural experiment, Urban form intervention, big data, public health, Physical activity, Social participation, Well-being, Equity, GPS, Accelerometer

## Abstract

**Background:**

Urban form interventions can result in positive and negative impacts on physical activity, social participation, and well-being, and inequities in these outcomes. Natural experiment studies can advance our understanding of causal effects and processes related to urban form interventions. The INTErventions, Research, and Action in Cities Team (INTERACT) is a pan-Canadian collaboration of interdisciplinary scientists, urban planners, and public health decision makers advancing research on the design of healthy and sustainable cities for all. Our objectives are to use natural experiment studies to deliver timely evidence about how urban form interventions influence health, and to develop methods and tools to facilitate such studies going forward.

**Methods:**

INTERACT will evaluate natural experiments in four Canadian cities: the Arbutus Greenway in Vancouver, British Columbia; the All Ages and Abilities Cycling Network in Victoria, BC; a new Bus Rapid Transit system in Saskatoon, Saskatchewan; and components of the Sustainable Development Plan 2016–2020 in Montreal, Quebec, a plan that includes urban form changes initiated by the city and approximately 230 partnering organizations. We will recruit a cohort of between 300 and 3000 adult participants, age 18 or older, in each city and collect data at three time points. Participants will complete health and activity space surveys and provide sensor-based location and physical activity data. We will conduct qualitative interviews with a subsample of participants in each city. Our analysis methods will combine machine learning methods for detecting transportation mode use and physical activity, use temporal Geographic Information Systems to quantify changes to urban intervention exposure, and apply analytic methods for natural experiment studies including interrupted time series analysis.

**Discussion:**

INTERACT aims to advance the evidence base on population health intervention research and address challenges related to big data, knowledge mobilization and engagement, ethics, and causality. We will collect ~ 100 TB of sensor data from participants over 5 years. We will address these challenges using interdisciplinary partnerships, training of highly qualified personnel, and modern methodologies for using sensor-based data.

## Background

The design of urban form or the built environment has been associated with people’s physical activity [1], social participation [2], and well-being [3]. The built environment also plays an important role in shaping social inequities based on differential access to resources and exposures to risk conditions [4]. Evidence about the potential impact of urban form changes is needed. In Canada, the federal government recently pledged $125 B for infrastructure projects over the next 10 years. While such investments may translate into positive health benefits, primarily due to increases in physical activity [5], natural experiment studies on urban form changes to date have been scarce.

Physical inactivity is the fourth leading cause of death globally responsible for some 9% of premature mortality and 6–10% of coronary heart disease, diabetes, and breast and colon cancers [6]. If the population added just 10 min of physical activity per day substantial health benefits at the population level would be achieved [7, 8]. Interventions at the environment and policy levels are considered key to achieve such population-level changes [6]. Lack of social participation has, in turn, been linked to myocardial infarction, stroke, cancer, and diabetes [9–11], especially among older adults and vulnerable populations. Well-being is an essential resource for individual’s health and is considered as a measure of societal progress [12]. The characteristics of the urban environments represent an interesting strategy to optimize the well-being of the population through changes in urban form [13–15].

Population and public health is committed to measuring and modifying urban form to support physical activity, social participation, and well-being, as well as reduce social inequities in health [16]. Urban form infrastructure [17], including cycling infrastructure [18] and traffic calming [19], is less likely to be implemented in low income areas, potentially exacerbating existing social inequities in health. Thus, stakeholders including urban planners, public health officials, and policy makers could benefit from more rigorous evidence to identify urban interventions that enhance population health and reduce social inequities in health [20, 21].

Natural experiment studies have been proposed as an important method to study how the large investments in infrastructure could cause health benefits related to physical activity, social participation, and well-being. Natural experiment studies are of interest because they can improve causal claims and lead to relevant and timely policy recommendations [22]. However, in a review of natural experiments evaluating policy and urban form changes on obesity-related outcomes, of the 17 studies that examined impacts on physical activity [23] only four had a strong study design (i.e., longitudinal studies with a comparison group), and most measured outcomes through self-reports or direct observation (e.g., cyclist counts), which have known limitations. Nonetheless, technological innovations related to mobile sensing can overcome some of the methodological limitations of natural experiment studies. These include the ability to reliably measure individual physical activity and daily mobility patterns using mobile sensors [24, 25].

Funded by the Canadian Institutes of Health Research, the INTErventions, Research, and Action in Cities Team (INTERACT) is a pan-Canadian collaboration of interdisciplinary scientists, urban planners, and public health decision makers advancing research on the design of healthy and sustainable cities for all. In partnership with cities and citizens, INTERACT is collecting big data to deliver timely public health evidence about influence of urban form interventions on health, well-being, social participation, and social inequities in these outcomes. Our objectives are to use natural experiment studies to deliver timely evidence about how urban form interventions influence health, and to develop methods and tools to facilitate such studies. Specifically, we will conduct natural experiment studies of urban form changes in four Canadian cities, with the aims to:

1. Understand Context: to characterize the context for changes in urban form, from an intersectoral perspective.
2. Measure Change: to refine measurement of changes in urban form exposures and outcomes using innovative methodological tools that integrate mobile sensing and Geographic Information Science.
3. Analyze Impact: to determine the impact of changes in urban form on physical activity, social participation, well-being, and related social inequities.
4. Mobilize Knowledge: to guide decision making on healthy urban planning and enhance training and capacity for urban form research in Canada using our evidence, methods, and tools.

## Conceptual framework

**Fig. 1:**
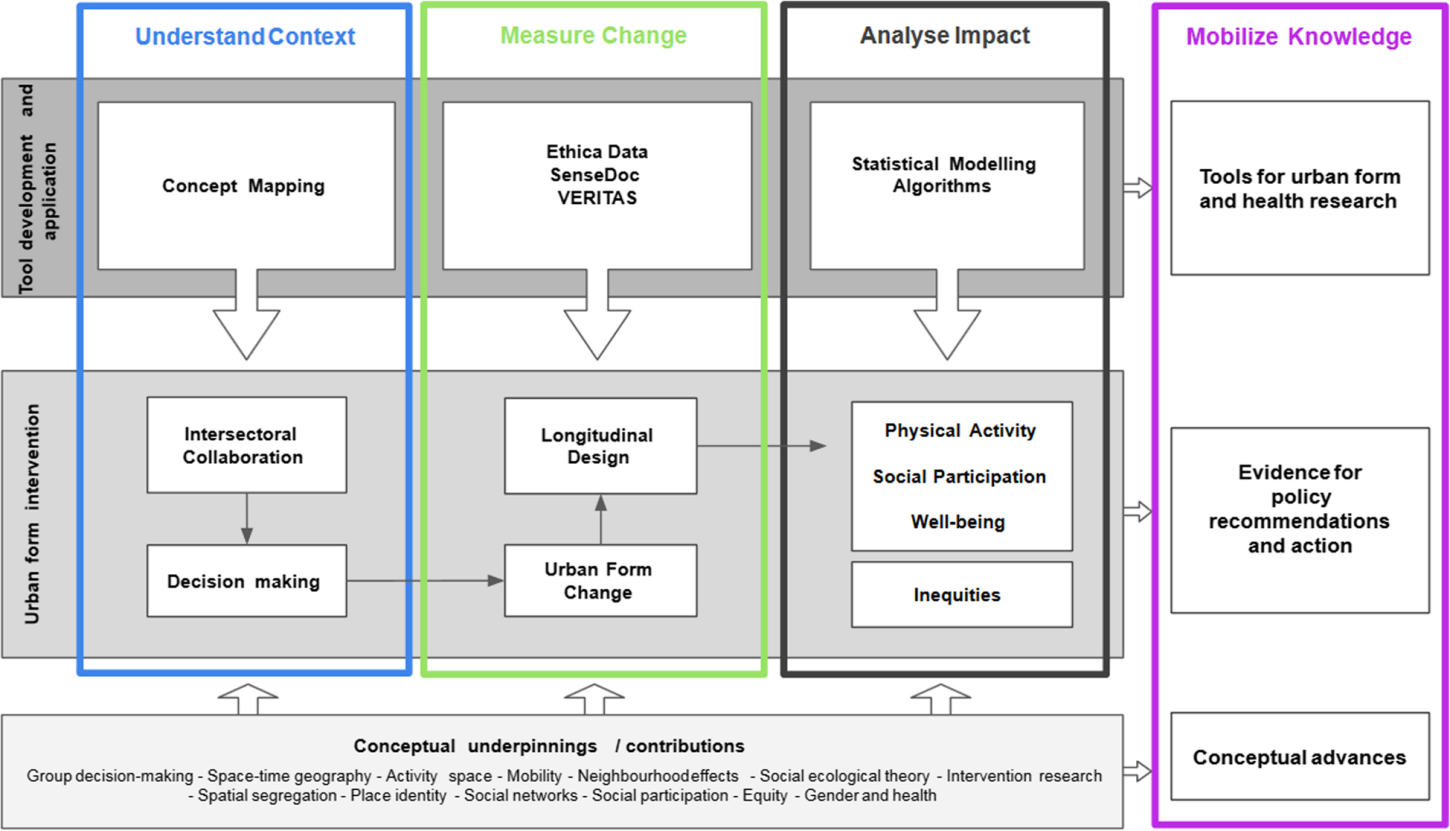
Conceptual framework for the INTERACT studies

The INTERACT conceptual framework (Fig. 1) is organized along four aims that emerged from a concept mapping activity among the research team and stakeholders, and reflect the intersectoral perspectives at play in urban form and health. Each aim includes a series of methodological tools that the INTERACT team will refine or develop (see grey box).

## Methods

### Interventions and cities

The INTERACT project will examine the potential health impacts of interventions in four Canadian cities.

**Victoria (All Ages and Abilities (AAA) Cycling Network)**, a commitment of $14.3 M to implement an All Ages and Abilities (AAA) Cycling Network, starting with the implementation of a 5.4 km grid in the downtown core. Protected bike lanes, off-street paths, and low-volume shared-use routes will connect every neighbourhood and regional trails [26].

**Vancouver** (**Arbutus Greenway),** a 9 km rail corridor the City of Vancouver purchased in 2016 for $55 M, which is being developed into a continuous walking and cycling corridor through prime real estate connecting South Vancouver to False Creek [27].

**Saskatoon (Bus Rapid Transit (BRT)),** a $66 M investment in a 22 km Saskatoon BRT system along three major roadways. BRT is an enhanced bus system, operating on bus lanes or other transit ways to combine the flexibility of buses with the efficiency of rail [28].

**Montreal (Sustainable Development Plan 2016–2020),** reflects a total investment over $100 M, is a comprehensive sustainability plan which includes urban form changes initiated by the city and approximately 230 partnering organizations that will be implemented. We will specifically evaluate how traffic calming measures, changes in transportation infrastructure, place making, and greening program influence our outcomes of interest [29].

### Design

#### Governance and partnerships

**Fig. 2:**
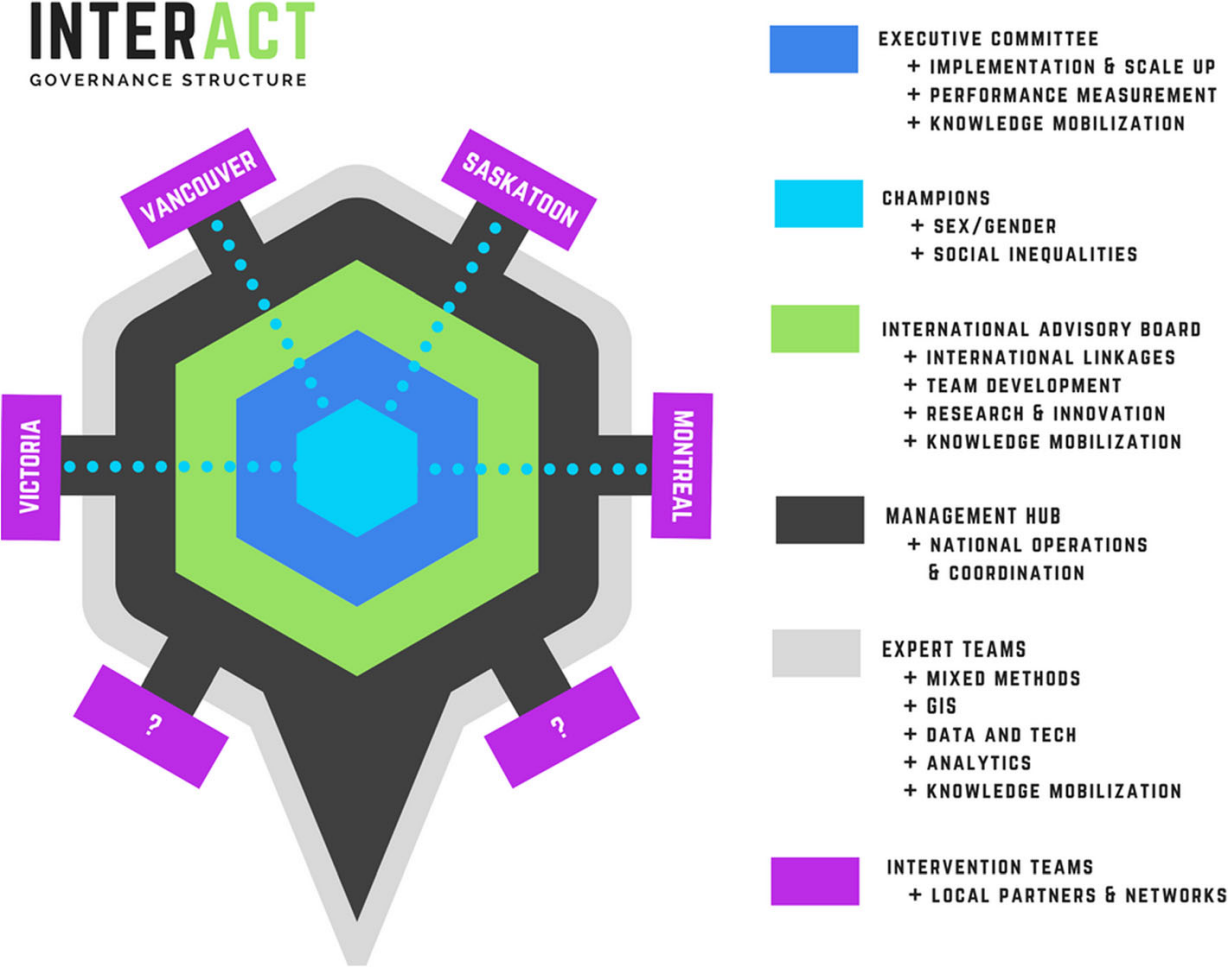
INTERACT governance structure

INTERACT is a pan-Canadian, bilingual team working collaboratively with local and national partners. The INTERACT Charter sets out the purpose, guiding principles, and inner workings of our team. While there is no one-size-fits-all approach to governance, having a clear and agreed upon structure showing how groups, objectives, and functions fit together to achieve its mission is an important element of successful research teams [30]. INTERACT’s governance structure includes six intersecting levels: management hub, executive committee, international advisory board, champions, expert teams, and intervention teams (Fig. 2). The intervention teams are intersectoral action-oriented teams guiding site-specific activities; the governance structure highlights that additional INTERACT sites may be added to the four existing study sites, given new funding or opportunities.

#### Intervention context

We will measure the intersectoral context of the interventions and their implementation using concept mapping. This method documents and synthesizes views and perceptions of groups and identifies convergent and divergent views between subgroups [31]. Concept mapping will be used to describe diverse stakeholders’ perspectives on the factors that influence the success or failure of the INTERACT urban form interventions prior to the interventions.

#### Study design

**Fig. 3:**
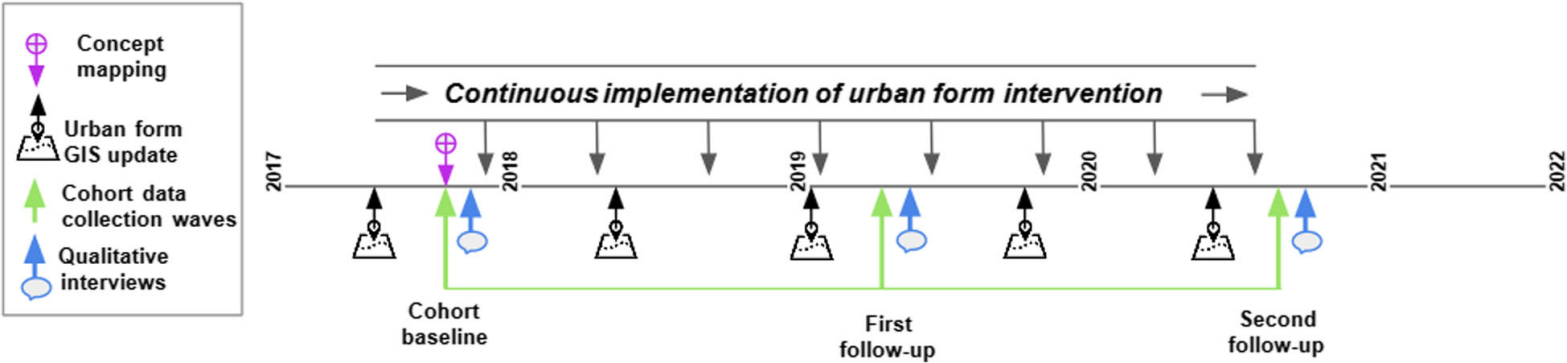
Example data collection timeline for an INTERACT study

The INTERACT study design is a mixed methods prospective natural experiment study that will follow adult participants (aged 18 and older) which will be implemented in each of four cities across five years. Our research approach collects comparable data between sites and also allows site-specific research activities. Quantitative data will be acquired over three data collection waves, baseline prior to or over the course of the intervention launch, and two follow-up time points. Timing of follow-up will be based on city context and each city’s intervention timeline. Figure 3 represents typical data collection waves and intervention implementation.

We will use qualitative methods to explore participants’ experience of urban form change as it pertains to physical activity, social participation, and well-being. These may include semi-structured individual interviews, or go-along methods, depending on the site. Detailed observation notes and videos of the interventions will be taken at each site.

#### Sampling plan

In Victoria, Vancouver, and Saskatoon we will aim to recruit 300 adult participants per site at baseline. We will use an open cohort methodology and recruit new participants to ensure a consistent sample size in follow-up waves. We will recruit samples representative of the age, gender, and socio-economic status specific to our exposure criteria in each city. In Montreal, where the exact location and timeline of interventions is not known a priori, meaning changes could be implemented anywhere throughout the city, 3000 adult participants will be recruited. Table 1 presents the inclusion criteria for each city, which varies based on the intervention being implemented in terms of target user and geographic scale. The exclusion criteria in all cities are being less than 18 years old, not being able to read or write English (or English or French in Montreal) well enough to answer an online survey and any intention to move out of the region in the next two years.

**Table 1:** Inclusion criteria for participants in the four INTERACT cities

City	Inclusion Criteria
Victoria	• Living in the Capital Regional District • Bicycle at least once a month in the City of Victoria.
Vancouver	• Living within 3 km of the Arbutus Greenway • Leave home at least once a week
Saskatoon	• Living in the City of Saskatoon • Take the bus in Saskatoon at least once per month or live within an 800 m buffer of the Bus Rapid Transit lines
Montreal	• Living in Montreal Metropolitan Area (Island of Montreal, Laval, Longueil, St-Lambert, Brossard) • Leave home at least once a week

### Data collection

**Fig. 4:**
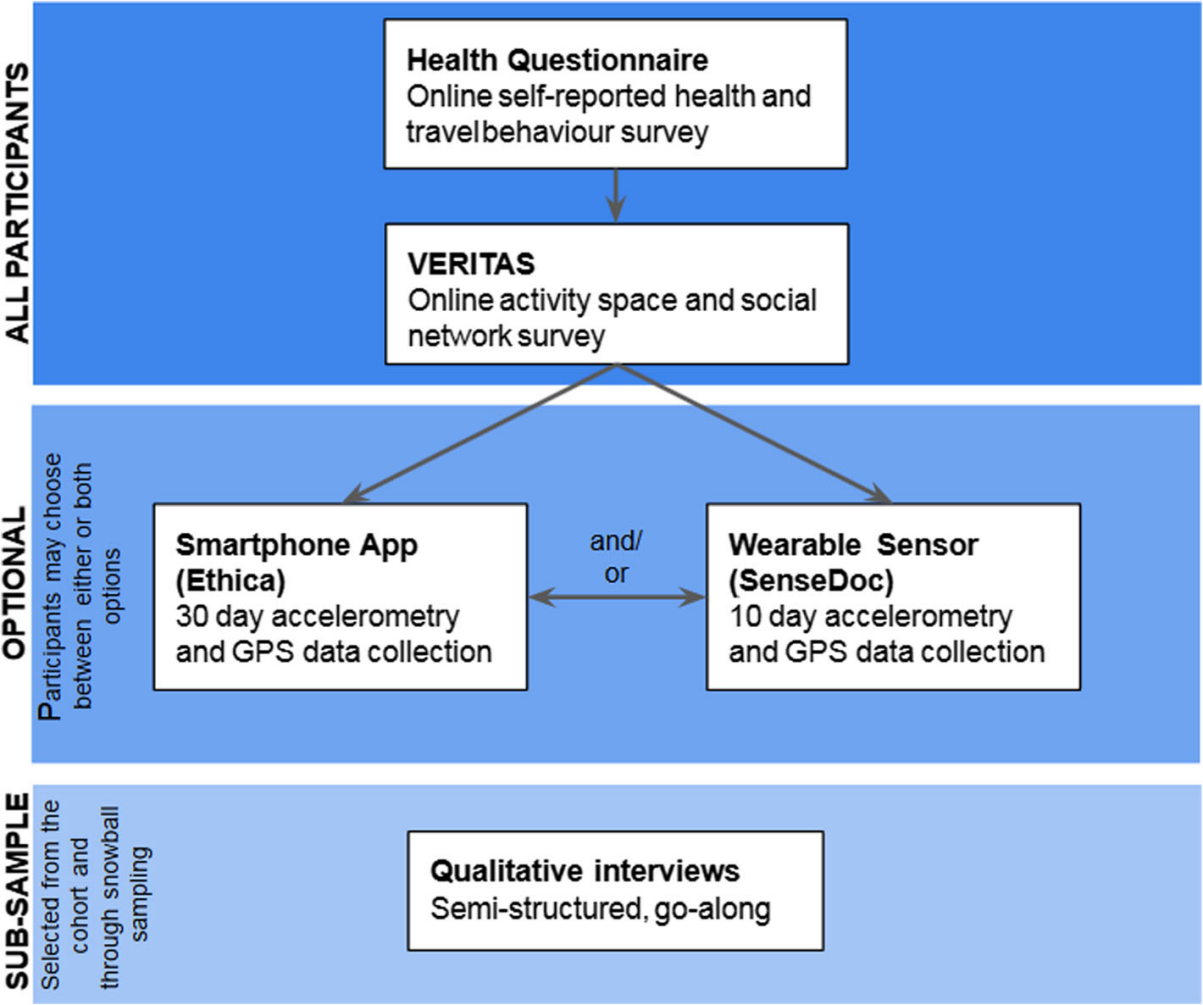
Participant flow diagram for INTERACT studies

We require at a minimum that participants complete an online survey, and in addition they may choose to install an app on their mobile device, wear a mobile sensing device, participate in qualitative interviews, or any combination of the above. For participants who may be interested in the study but do not have internet access and a computer we will provide the opportunity to complete the surveys in person or over the phone with a research assistant. Figure 4 shows the flow diagram for potential participants in the INTERACT studies. Ethics approval has been received from the ethics boards of Simon Fraser University, the University of Saskatchewan, the *Centre de Recherche du Centre hospitalier de l’Université de Montréal,* and Memorial University of Newfoundland.

#### Quantitative data collection methods

We will use online surveys and two mobile sensing methods to collect quantitative data. Online surveys will be used to measure health, physical activity, social participation, travel behaviour, well-being, and activity spaces. The surveys are posted on the team website. We will use the Visualisation, Evaluation and Recording of Itineraries and Activity Spaces (VERITAS), an online map-based survey that collects data to measure participant activity spaces and social networks [32].

The mobile sensing tools used to collect physical activity and spatial location data will be Ethica Data [33], an iOS and Android smartphone app, and SenseDoc [34] a research grade accelerometer device. Participants will be asked to install Ethica Data on their phone for 30 days and/or to wear a SenseDoc for 10 days. We will combine accelerometer and Global Positioning System (GPS) data in order to measure location-based physical activity and infer transportation mode [35, 36].

We will use the Ethica Data smartphone app to measure well-being using ecological momentary assessment. We will use the validated Short Mood Scale [37], which measures three dimensions of affective states: valence, calmness, and energetic arousal.

#### Qualitative data collection methods

We will use qualitative approaches to explore participants’ experience of urban form change as it pertains to physical activity, social participation, and well-being. We will recruit approximately 20 participants per site, by selecting participants from the cohort and supplementing with criterion-snowball sampling [38] and respondent-driven snowball sampling [39]. Our approach will consist of a semi-structured interview to gather descriptions of individual experiences of urban form, in general and with respect to the specific intervention. Qualitative data collection will also involve go-along interviews in certain cities [40].

#### Exposure measurement

Measuring exposure is crucial to correctly estimate the impact of interventions on our outcomes of interest [41]. Through partnerships with knowledge users we will use existing resources such as local city data, and open data to collect exposure data. Assigning individuals to exposed and unexposed groups in natural experiment studies is challenging, both from a conceptual and methodological standpoint [41]. As people move through urban environments, exposure is a dynamic rather than a static process. We will measure absolute and relative exposure to the urban form intervention using at least three methods: home location, activity space, and daily GPS path [42, 43]. Our approach will allow us to compare static and dynamic methods for measuring exposure and contribute to the discussion of how to define and measure exposure to urban form.

#### Power

Sample size calculations for natural experiment studies have little precedent [44]. Longitudinal and cross-sectional mobile sensing studies have used sample sizes ranging from 34 to 550 [58–62] and 28 to 1300 [63–70], respectively [45–51]. We used the EpiTools cohort study sample size calculator (http://epitools.ausvet.com.au/) with parameters based on the physical activity literature. Assuming repeated measures, power of 0.8, alpha of 0.05, expected incidence in the unexposed of 0.15, and expected relative risk from 1.5 to 1.8, we estimate that between 422 and 683 participants will be required to detect intervention effects. However, the implications for power with thousands of repeated measures per participant are still not well defined in the literature [52].

#### Measures

We have based our methodology on existing knowledge in order to develop the data analysis plan in as much detail as possible; however, the INTERACT project will require the adoption of new methodologies currently in development. As a team we are committed to open science and will post code and analyses online (https://github.com/teaminteract).

#### Physical activity

We will assess physical activity both through self-report (questionnaire) and objective (Ethica smartphone app and SenseDoc devices) measures. With objective data, we will create measures of total volume of physical activity, cut-points for physical activity intensity levels, and activity type (e.g. sitting) [53]. To our knowledge, there has been limited development of cut-points of physical activity intensity using smartphones [54]. Studies using raw accelerometer data have employed a number of different machine learning methods including artificial neural networks to develop physical activity indicators from smartphone data [55, 56].

#### Well-being

We conceptualize well-being along hedonic and eudaimonic dimensions. Hedonic well-being focuses on the presence of positive affect, the absence of negative affect and the cognitive evaluation one’s satisfaction with life. Eudaimonic well-being refers to optimal psychological functioning [57]. We will measure intra-daily variations in affective components of hedonic well-being via Ecological Momentary Assessment (EMA) using the Short Mood Scale [58]. INTERACT will measure cognitive components of hedonic well-being via the health survey using the Subjective Happiness Survey [59] and the eudaimonic well-being using the Personal Well-Being Index (PWI-A) [60]. More in-depth feelings of both dimensions of well-being will be explored with the qualitative research component.

#### Social participation

We will measure social participation using the Sense of Community Belonging questions from the Canadian Community Health Survey [58], the Social Cohesion and Trust question (Collective Efficacy) from the Project on Human Development in Chicago Neighborhoods study [61], and the question on trust and reciprocity from the General Social Survey [62]. The map-based VERITAS survey will further allow us to measure participants’ social network size and strength. Finally, deeper experiences with social participation related to urban form will be explored within the semi-structured interviews.

#### Transportation mode detection

To examine changes in transportation based physical activity, we will need to develop or apply existing methods that will allow us to predict transportation mode and activity locations. There is considerable research currently being produced in this area [63, 64]. We will apply those methods that provide sufficient accuracy for our needs, but will develop or modify new analytic techniques for trip mode detection when necessary.

#### Geographic Information system (GIS) exposure

Measuring and understanding exposure to and changes in the urban environment is crucial for our work. Our team has also reviewed GIS measures in the literature to characterize green space, park access, land use mix, transit access, and bike infrastructure, in order to identify optimal measures for our project. There are currently a number of methods being developed with potential application to our work [64–66]. For instance, our team members have used network kernel density estimation to examine changes in cycling collisions [67]. This method could be generalized to different types of spatial data (ie. polygon and polyline) that will be necessary to understand changes in exposure over time.

### Data analysis

#### Intervention analysis

The potential for natural experiment studies to improve causal claims about intervention effects has received considerable attention [68, 69]. There are a number of potential analysis methods including, regression discontinuity [70], difference in differences [71] and interrupted time series [72] that will be adopted to examine the impact of the natural experiments. As INTERACT data collection is longitudinal within each intervention city, our planned method for impact assessment will be interrupted time series. Analyses applying regression discontinuity and difference in differences are also possible depending on the research question. Analysis methods for causal inference are advancing quickly [73] and we will implement new methods that are relevant and applicable to INTERACT research questions.

#### Qualitative analysis

Interview recordings will be transcribed verbatim by a professional transcription agency. Data will be analyzed using framework analysis [74], an appropriate analytic approach for qualitative studies with specific questions, a pre-designed sample, and issues identified a priori. We will create a broad coding framework aligned with the aim of each interview question. During a second cycle of coding, we will apply a focused coding procedure to the broad coding framework. In a third and final cycle, we will simplify the coding framework into thematic categories, and reach consensus on this final codebook. The study team will meet regularly during data collection to discuss coding and the preliminary findings, as a step to ensure rigour.

#### Equity analysis

We will examine how intervention impacts are socially and spatially patterned using an equity analysis approach [75]. This approach focuses on factors that impact the spatial distribution of exposure, the spatial distribution of populations, population vulnerability, and the interaction between these factors.

## Discussion

INTERACT is a pan-Canadian collaboration of interdisciplinary scientists, urban planners, and public health decision makers advancing research on the design of healthy and sustainable cities for all. Seeing the city as a living laboratory, we are using innovative tools such as mobile technology to measure the impact of changes in urban infrastructure on people’s physical activity, social participation, and well-being, and inequities in these outcomes. With INTERACT’s aim to advance the evidence base and generate new tools, the project will face diverse challenges. We highlight considerations related to big data, knowledge mobilization and engagement, ethics, and causality below.

### Big data

We estimate that INTERACT will collect approximately 100 terabyte (TB) of raw data during the study period. The volume and variety of data make data security, storage, and processing a crucial consideration for the INTERACT study. We plan on partnering with both the Centre for Health Information and Analytics at Memorial University and Compute Canada for data infrastructure requirements. There are considerable training requirements for public health researchers and students in both data handling and machine learning methods [25]. We will develop training modules related to working with large data infrastructure, programming in Python and R, and methods for analyzing large spatio-temporal datasets.

### Knowledge mobilization

The INTERACT program is guided by a framework for sustained impact [76], which highlights the importance of early and sustained engagement with non-government organizations and policy makers nationally and locally. The meaningful engagement of intersectoral stakeholders throughout the research process will be guided by a governance model that prioritizes consultation and collective decision-making, and the equitable participation of knowledge users. For example, city decision-makers are integrated within intervention teams, positioning them to provide input on local research questions and methods, and to facilitate evidence-informed decision-making. We have a diversity of knowledge translation end products planned, including both applied and scholarly outputs relevant to municipalities, regional governments, health authorities, non-governmental organizations, academics, and citizens. One example is that we have adopted a process of rapid reviews that can inform research or policy at the local level. We also aim to enhance training and capacity for urban form research through open and wide dissemination of INTERACT’s evidence, tools, and methods to both practitioner and research communities.

### Ethics

There are ethical considerations for INTERACT, specifically related to the high precision location data collected from participants [77]. In natural experiment studies using mobile sensing it is important to recognize risks that are part of the intervention, and risks that arise through unintended circumstances during the study. Risks related to the intervention are unlikely in a natural experiment study because researchers do not control the intervention [77]. There are risks related to using mobile sensing data for health research including consent, privacy and confidentiality, mitigating risk, and vulnerable populations. We will mitigate potential risks by using plain language consent forms, having clear Frequently Asked Questions (FAQ) support for participants, updating participants about the data they are contributing, using secure protocols for data storage and access, and training research trainees and staff on data security and access [78, 79].

### Causality

Making causal claims about intervention effects is the primary objective of epidemiologic studies [80]. Regression discontinuity, difference in differences, and interrupted-times series are methods that can permit causal claims to be made about intervention effects in observational research, provided that the assumptions of the methods are met. A challenge of evaluating the impact of urban form interventions is that interventions and individual level exposures are changing in both time and space [81]. In contrast to strict interventions, which can be implemented from one day to the next, urban form interventions are implemented over longer time periods and often in phases. The nature of urban form interventions will require us to test multiple methods for defining exposure both temporally and spatially, and cautiously interpret our results. The on-going nature of the intervention implementation and confounding by other urban form changes will be a challenge for causal attribution [82].

### Limitations

INTERACT faces limitations related to sampling bias, control groups, intervention specificity, and context. Sampling bias may take two forms. First, vulnerable populations are less likely to participate in research in general and may be even less likely to participate in research involving mobile sensing, either because they may not have these devices or because the detailed location data collection may be considered additionally intrusive to these populations [78]. Second, we have not a priori identified control groups, either in the form of comparable cities or comparable unexposed groups, for our interventions. However, for some research questions, spatial exposure can serve to identify levels of exposure within the sample. Third, INTERACT will evaluate four different urban form interventions in four different cities. Both the conceptual and statistical identification of the hypothesized and actual intervention effects and which aspects of the intervention we are evaluating is challenging. There are several potentially relevant contextual features to consider including: inequities, public consultations, media campaigns, and political decision making. With only four cities included, we are using an ad hoc approach to consider how these and other contextual factors should be considered in our analysis.

INTERACT will harness big data to deliver timely public health evidence on the influence of real-world urban form interventions on physical activity, well-being, and social participation, as well as social inequities in these outcomes. We aim to understand context, measure change, analyze impact, and mobilize knowledge about how the urban form can influence health.

## Abbreviations

AAA: All Ages and Abilities
BRT: Bus Rapid Transit
EMA: Ecological Momentary Assessment
FAQ: Frequently Asked Questions
GIS: Geographic Information System
GPS: Global Positioning System
INTERACT: INTErventions, Research, and Action in Cities Team
PWI-A: Personal Well-Being Index-Adult
TB: Terabyte
VERITAS: Visualisation, Evaluation and Recording of Itineraries and Activity Spaces
